# *“We Can Do This!”*: The Role of Physical Activity in What Comes Next for Dementia

**DOI:** 10.3390/ijerph20156503

**Published:** 2023-08-02

**Authors:** Christopher Russell

**Affiliations:** Association for Dementia Studies, University of Worcester, Henwick Grove, Worcester WR2 6AJ, UK; c.russell@worc.ac.uk

**Keywords:** dementia, physical activity, person-centred, human rights, citizenship, relationships, agency, power, the everyday, leisure

## Abstract

There is increasing interest in physical activity as a response to the harm caused by dementia. The focus has been upon interventions to prevent or delay symptoms or to support people living with the condition to reminisce. Whilst this is welcome, there are other features inherent to physical activity that remain unrecognised or underutilised and, consequently, its full potential for good is unrealised. Most prominent is the ability physical activity has to enable participants to claim and sustain a place in the world through what they do, crucial to a context where the impact of dementia tends to annihilate this for those living with the condition. The article addresses this gap. In doing so, it presents key findings. These include (1) highlighting the fundamental importance of features such as person-centred care, human rights and social citizenship to enabling people with dementia to live lives of quality and (2) identifying synergies with these features and what physical activity can offer; for example, emphasising the value of bringing these together to illustrate how physical activity can contribute to enabling people with dementia to live lives characterised by quality, and the maintenance of their place in the world. The article concludes by suggesting what must come next to ensure physical activity can play the fullest role possible.

## 1. Introduction

This article focuses upon life with dementia and the role physical activity should play within it. It highlights how modern thinking about dementia (in particular, two dementia-related paradigms: person-centred care and support and social citizenship) aligns with insights garnered from contemporary scholarship about physical activity. Insight into this modern thinking is offered to illustrate ways in which physical activity can offer much to people living with dementia beyond improvements to physical health (important though these are) and how physical activity can contribute to enabling people with dementia to sustain their place in the world. Suggestions are made so that practice can be tailored to maximise the benefits such approaches can bring, and the article concludes by highlighting key areas for attention so progress can be realised. Firstly, however, dementia and physical activity are explained alongside a fuller rationale for what follows.

Dementia is an umbrella term used to describe a number of diseases or conditions that cause irreversible damage to the brain, with symptoms that inevitably progress over time. The nature of these will vary depending on the particular form of dementia causing illness; for example, Alzheimer’s disease (the most common form of dementia) is characterised by memory loss in relation to contemporary events [1,2]. Only very recently has any advancement been made with pharmacological interventions that might alter the progression of symptoms [3]. Possible breakthroughs relate solely to Alzheimer’s disease and are limited to intervention at early stages of the illness, with delivery available through intense and uncomfortable means (i.e., subcutaneously). The potential for malign side effects means that, in large parts of the world, regulatory bodies remain reluctant to approve use [4,5]. The fact that pharmacology has been unable to offer definitive responses to dementia has caused concern for individuals living with dementia, their families and health and care professionals [6]. In part, this is because, as symptoms progress, the ability of people to continue to play an agentic role in everyday life is corroded and eventually destroyed [7]. In addition, dementia is universally feared because of the impact it can have upon an individual’s sense of self and identity [6,8], with those living with the condition reporting feelings of stigmatisation and low self-esteem [9]. These matters, allied to the fact that very large numbers of people across the world are living with dementia or predicted to be living with it over the next fifty years [10], have made dementia a global public health priority [11].

With this context to the fore, research and scholarship have sought to comprehend best practice in areas related to the provision of care and support for people affected by dementia [12]. This has given rise to concepts characterised as “psycho-social” approaches. These include person-centred care [6] and personhood [8], which can contribute to enabling people with dementia to live lives characterised by quality by encouraging others to attend to individual need and maximising the quality of relationships inherent to the daily life of the person [13]. In recent times, these approaches have increasingly been regarded as inadequate in themselves because of the risk that they may foreground people living with dementia as patients rather than whole people, solely with needs to be met rather than lives to be led [14]. Critique has been driven, in large part, by people living with dementia, who, in the last 10 years or so, have advocated for enhanced recognition of the role of human rights in any discourse about dementia [15]. This has drawn upon, and simultaneously fuelled, an increasingly influential paradigm espousing social citizenship in dementia [16].

Citizenship is a comprehensive and complex phenomenon, the subject of study and interpretation over many years [17]. This dynamism is part of it, with rights and responsibilities of individuals and those of the state renegotiated as contemporary societal circumstances change [17]. In fact, defining citizenship should not be easy because such vitality of meaning is what enables it to be effective [18]. Social citizenship is the interpretation that has been adopted within the dementia context. It is a relationship or practice (as much as a status) whereby an individual living with dementia must feel free from discrimination and have opportunities to grow and participate in life as fully as possible [16]. Social citizenship involves the upholding of rights, with international frameworks—such as Article 30 of the United Nation’s Convention on the Rights of Persons with Disabilities—being seminal in enabling an authoritative rather than optional status [19]. Allied to this modern thinking is the necessity that people living with dementia should expect every actor with influence in their daily life (ranging from shop keepers to surgeons, depending on prevailing circumstances) to offer opportunities for the fullest engagement possible in “everyday life”. The nature of “everyday life” is seen as fundamental to social citizenship, with the mundane components of the everyday being the context in which people living with dementia manifest their ongoing place in the world [17].

Physical activity is defined as any intentional movement produced by the skeletal muscles that results in increased energy expenditure [20]. The broad nature of this interpretation means it is capable of being part of everyday life. Sadly, however, physical activity is not routinely on offer to people living with dementia [21]. There are many reasons for this, with one of the most prominent being that knowledge of what works well in terms of the provision of physical activity is limited [22]. However, as will be shown, physical activity is well placed to align with contemporary thinking about dementia because it can be used to harness elements of person-centred support and contribute to the social citizenship of people living with dementia. For these reasons, it should, therefore, be included as a fundamental part of the ongoing response to dementia.

## 2. Responses to Dementia and the Part Played by Physical Activity

There is increasing and global interest in what physical activity can offer to people living with dementia [23] because of its contributions in several different ways; for example, via potentially averting, holding up or improving symptoms of dementia [24,25], with evidence that suitable interventions may slacken the rate of disease progression [26]. In addition, within the field of dementia reminiscence, physical activity has been employed to stimulate memories of times past in the lives of individuals; for example, through use of Irish dancing [27]. However, what is proposed here, a focus upon person-centred and social citizenship approaches in dementia, builds upon and moves beyond this by highlighting how additional benefit can be realised through physical activity supporting and promoting one’s place in the world or in the everyday lives of participants with dementia. Furthermore, such understanding will enable those offering opportunities for physical activity to tailor what they do to enhance its ability to contribute even more than it already does, this being especially important because of the lack of reliable and accessible pharmacological responses to dementia. In the following paragraphs, therefore, person-centred and citizen approaches are explained in detail. Key aspects are identified that, as will be indicated, hold relevance for those interested in adapting approaches to physical activity for individuals living with dementia.

Turning first to person-centred support, over the last 25 years, there has been a move from considerations of dementia as primarily a medical condition to something recognised as affecting all aspects of a person’s experience of life [8]. Fundamental is the fact that, as every person is unique, everybody’s experience of dementia is different. Taking a person-centred approach therefore means ensuring that everyone living with dementia is recognised in terms of their life story, abilities and skills, personality and physical health rather than being defined only by the damage to cognition caused by dementia [6,12]. Furthermore, to facilitate person-centred approaches to care and support, what is known as “personhood” must be promoted. This is defined as “a standing or status that is bestowed upon one human being, by others, in the context of relationship and social being. It implies recognition, respect and trust” ([8], p. 8). Thus, the relationships an individual living with dementia has with others and the quality of those relationships in terms of enabling that person to sustain their feeling of self and identity matter. If these are not of sufficient quality, person-centred support cannot be sustained.

For the best part of a quarter of a century these ideas have formed the foundations of approaches to care and support in dementia [6]. However, person-centred approaches alone are insufficient to enable people with dementia to live lives of quality [14]. Emphasis by clinical services upon earlier diagnosis [28] has enabled more people living with dementia to articulate what matters to them in everyday life [29]. Through accounts of people themselves, and via allied research and scholarship, has come the call for human rights and social citizenship to be prioritised in ways not represented within person-centred approaches. For example, Bartlett and O’Connor [14], writing in 2007, highlighted that the structural nature of societies and the agency held by people living with dementia also needed to be taken into account. Thus, people living with dementia should be recognised as individuals whose experience of life is informed by their gender, class, ethnicity, sexuality, ability, and age [17].

A focus upon human rights is required because person-centred approaches alone fail to affirm the ongoing status of people as having power to influence their lives [30]. This, in turn, highlights the need to promote the ongoing place in the world of people living with dementia as citizens [17]. All too often, people lack agency [31] or indeed opportunity to demonstrate this, and later it will be argued that physical activity is well positioned to redress this. However, before addressing this directly, more detail is required relating to the nature of citizenship approaches in the context of dementia. This concerns elements that contribute to the foundations of this article’s core argument: that modern thinking about dementia (aligned with insights garnered from contemporary scholarship about physical activity) illustrates how physical activity can contribute to enabling people with dementia to live lives characterised by quality and sustain their place in the world.

Firstly, the nature of everyday life with dementia is now viewed as crucial. This is because it is here that the practical conditions for the participation and influence of individuals occur [17]. Thus, as Nedlund and colleagues advocated in 2019 [17], there is a need to take the ordinary seriously. Furthermore, as Butchard and Dunne [29] point out, the human rights of people living with dementia are liable to violation on an everyday basis. The example they highlight is the “task-driven” nature of care, removing the opportunities many people living with dementia have to influence what goes on or to express their preferences. Instead, social citizenship approaches advocate that people living with dementia should be enabled to explain or demonstrate what they wish to happen on an everyday basis and thus enact their human rights. As will be suggested later, physical activity provides a unique ability for individuals living with dementia to demonstrate this.

Secondly, the quality of relationships matters in ways extending beyond those identified within person-centred support. So, for example, Sabat [32] argued there should be no “us” and “them” when considering life with dementia. He advocated for approaches supportive of “shared humanity” ([32], p. 12). This theme has been built upon [17] with the suggestion that citizenship-based relationships should foster feelings of belonging, with a focus upon recognition of shared experiences within communal care settings, for example. Other people play a significant role in affirming the integrity of self-worth for people living with dementia through their actions and behaviour towards them [33]. Thus, those people, key within the lives of each individual, can contribute to the citizenship of a person through relationships characterised by shared humanity, a sense of belonging and affirmation of an individual’s self-worth [17,32,33].

How this might occur is related to a third point, which is that, in terms of recognition, it is acknowledgement by others of the agency held by the person living with dementia that matters most [34]. This is congruent with a “strengths-based approach” [32] in the dementia context, which prioritises abilities a person retains rather than those they may have lost as a result of the illness. Nedlund and colleagues [17] characterised this as shifting the focus from that exclusively upon care towards understanding that people can and must retain power. This should pertain to people at an advanced stage of dementia too and in such circumstances can be manifested via displays of “embodied agency” [34], where people employ actions to express their will if no longer able to do so verbally. As a result of the harm caused by advanced dementia, these can be subtle movements or gestures offered by individuals through what have been described as “micro-gestures” [17]. Those who may be supporting people with advanced dementia (for example, paid or family carers) must assist them by knowing them well and being able to understand their significance. This is “relational citizenship” [35], where individuals use their bodies to express their place in the world. The opportunities afforded for this by physical activity are clear. Unlike other leisure-based activities, such as singing or art, which potentially afford similar benefits in terms of communality and presence to people living with dementia, physical activity uniquely and by its very nature offers opportunity for individuals to employ their bodies to achieve such constructive outcomes. It is noteworthy also that relational citizenship in the dementia context is based upon the premise that people must be active partners in their own care. Thus, agency, power and how these are manifested by individuals and recognised by others form cornerstones of modern approaches to citizenship in dementia.

A rights- and citizenship-based approach is founded upon ethical concerns for social justice, fairness and equality. With regard to dementia, this has been described as “a discourse of hope” ([30], p. 18). That physical activity has a key role to play enhancing the social citizenship of people living with dementia is, therefore, exciting and positive. With these factors in mind, and drawing upon the discussion so far, the article now turns to the role of physical activity in everyday life with dementia.

## 3. The Role of Physical Activity in Everyday Life with Dementia

Physical activity is an inextricable part of the everyday. This is reflected in numerous studies exploring physical activity in the context of life with dementia (as will be shown below). Thus, it is well placed to form an essential part of the response to the condition. For example, a study by Olsen and colleagues in 2015 [36], which had as its basis a high-intensity functional exercise programme for those with dementia living in a nursing home, found there was much participants valued related to the everyday. Thus, individuals reported that participation enabled them to feel “…more like a normal person” ([36], p. 6). This was related to physical activity having felt relevant to the everyday life of participants throughout earlier times and this feeling extending into daily living within the context of the nursing home; for example, enhancing one’s energy to engage within social discourse and offering the chance for such opportunities to happen in the first place via the provision of the physical activity. Writing in 2022, Telenius and colleagues [37] highlighted similar features. Their study involved interviews with people living with dementia to explore their experience of physical activity. Notable here were participant reflections about physical activity giving content and structure to their days. “Everyday” activities, such as mowing the grass, were highlighted as examples. The significance of the seemingly mundane had deeper consequences, however, when considered against what was discussed about agency and power. Participants noted, for example, that such routines enabled them to exert feelings of control within daily life that would otherwise be missing.

Of course, what individuals choose to do with the everyday must not be prescribed. This article champions person-centred approaches based upon rights and citizenship within everyday contexts, and people not only have a right to choose what they do in later life but they also have the right to choose to do nothing very active if this is their wish [38]. However, because physical activity is so diverse in nature, it is well placed to embrace a range of activities that may align with the preferences individuals have for activity in their everyday lives. A study by Burke and Jones [39] is illustrative. It focused upon experiences of people living with dementia within sheltered housing and care homes but, unlike the research conducted by Olsen et al. [36], investigated reflections offered by participants engaging in “low threshold” sports programmes. This placed “minimal demands” ([39], p. 2) in terms of what was required or expected of individuals. Facilitators were not themselves highly trained sports professionals or therapists; instead, they were provided with training in the interventions in order to offer them with confidence. Participants enjoyed what they did, and it was noted that provision could be sustained over the long term because it was “open to all” and sociable in nature. This low-threshold physical activity had the potential to act as a gateway to further opportunities for individuals ([39], p. 8).

Thus, physical activity has an important role to play in what is on offer to people living with dementia as part of everyday life, bringing benefits in terms of sustaining an individual’s place in the world through what they do and experience. Other studies have affirmed this, including that by Hartfiel et al. [26], which, although investigating outcomes of an organised exercise programme for people living with dementia, highlighted how the formation of habits revolving around physical activity was beneficial for individuals. For example, one participant reported that, after engaging in the programme, he had gone on to complete nearly 40 park runs. The everyday is about tailoring physical activity to what is relevant to the person living with dementia [24]. This can be highly structured and organised or it can be as mundane as shovelling snow [36]. Links here to the earlier discussion about the need to pay attention to person-centred approaches are clear.

The nature and quality of relationships held by people living with dementia were identified (above) as positive and fundamental in enabling individuals to sustain their place in the world. Physical activity has been shown to offer opportunity for such constructive relationships. For example, Olsen and colleagues [36] found that it was the interactions amongst participants that contributed significantly to those individuals realising the outcomes they wished to achieve from the activity, here acting as role models for each other, demonstrating what was possible and motivating each other to participate. As one respondent said: “…we can do this!” ([36], p. 6). Collaboration is seen as a powerful and positive enabler in sustaining one’s place in the world in the context of life with dementia [17,33]. This involves not only a sense of belonging, which, as has already been highlighted, is key to the sort of empowerment at the heart of this discussion, but also feelings of agency and of holding power in relation to what is being enacted. Confidence to engage in such collaborative actions can come from within the group itself [26]. However, there are nuances and skills that facilitators must be aware of in order to foster collaboration. These include the willingness of those nominally charged with providing physical activity to enable participants living with dementia to lead and facilitate such activity at times or throughout the process, if this is their wish [24]. The provision of physical activity in such ways can have beneficial outcomes for staff and volunteers too; for example, increased optimism and positivity have been reported throughout settings where this has taken place [24]. When this operates successfully, encounters by those living with dementia with staff have been found to be characterised by vitality rather than detachment [36]. These are important attributes linked to the provision of physical activity within a context where so often the wellbeing and motivation of the dementia care workforce is not found to be so buoyant [40,41].

This discussion has shown that physical activity can act as an enabler of agency and power for people living with dementia. The significance of this to the facilitation of an ongoing sense of place in the world for individuals living with the condition, however, means that a fuller examination is required. Physical activity can offer the opportunity for individuals to test themselves physically and competitively. Whilst the definition adopted in this article does not prioritise this, it is widely accepted that it can do so. How much individuals living with dementia can achieve for themselves through engaging in physical activity has been found to matter [36]. For example, there are factors such as others having expectations of what individuals might achieve. Such an approach is unlikely to suit everyone; however, people living with dementia report that stereotypical depictions among their peers can lead to their own feelings of stigmatisation [42]. Rather than an overt focus upon goal setting, consideration should be given to other related features found within relevant research. These could include, for example, giving attention to the value that people living with dementia report being invested with and their reports of being noticed through what they did [36]. People living with dementia have also reflected upon the benefits of feeling they “still measured up” ([21], p. 4) through the ability to test this feeling through use of their body. This was found also to involve a sense of mastery over a particular activity, with self-efficacy (the ability to achieve for oneself) being especially prized [21].

However, care needs to be taken when exploring this aspect of the discussion. Throughout, the importance of person-centred approaches has been advanced to run alongside the merits of other features. This is a moment to reflect upon the significance of this because what will suit one person will not be for another. Indeed, a person’s preference may vary from one engagement in physical activity to the next [24]. A sense of agency and power can be achieved by people living with dementia participating in physical activity in other ways; for example, as highlighted by Olsen et al. [36], through activity that is challenging but fun. The value individuals report of being able to look forward through ongoing participation over time must also be considered [36]. Conceptualisations of leisure have been applied in such circumstances within the dementia context. For example, “serious leisure” [43] has been used to explain how, through physical activity, individuals have been empowered to acquire and enact skills via the regular progression of activities that are substantial, of interest and fulfilling [44]. If activities feel relevant to the person involved, and if the experiences at the time matter to them, then this may be enough to offer that individual the sense of agency and power required [24].

## 4. What Should Happen Next?

Physical activity is part of the everyday, particularly with its widely accepted, broadly drawn definition (cited above) to the fore. Thus, it needs to be taken seriously as a means to enable the sustenance of the place in the world of individuals living with dementia. Scholarship from the fields of dementia and physical activity has affirmed this by making the links between physical activity, dementia and the everyday. However, questions remain about the way forward. These relate to matters highlighted in the preceding discussion, but there are also elements that the article has been unable to consider fully enough. In this section, the question of what should happen next is posed. The answers to this and each of the queries raised as part of it will be fundamental to enabling physical activity to realise its fullest potential within the context of life with dementia.

For example, it is not yet clear what skills and attributes are required to offer physical activity to people living with dementia. The articles cited here are helpful in progressing understanding. However, there remains much from the milieu of dementia scholarship and practice that could still contribute. For example, the pioneering work on person-centred practice in dementia [8] has been advanced by others [7] so that person-centred approaches could be understood in theoretical terms and underpin care and support for those living with dementia. In turn, this has enabled good practice to be distilled into resources to enable services and organisations to operationalise what they do (for example, see the “Care Fit for VIPS” framework at https://www.worcester.ac.uk/about/academic-schools/school-of-allied-health-and-community/allied-health-research/association-for-dementia-studies/ads-education-and-research/free-resources/care-fit-for-vips.aspx accessed on 1 February 2023). There is a need for an overarching theory expounding good practice in the facilitation of physical activity for people living with dementia. With initiatives such as social prescribing gaining traction, this would seem timely, especially as the evidence base underpinning it remains uncertain [45].

Who is best placed to be involved in the delivery of physical activity also remains opaque. The authorities drawn upon in this article have not enabled a conclusion to be reached. Some advocated for the involvement of specialists and therapists. For example, Olsen et al. [36] opined that expert knowledge about the aging body is essential. However, as noted above, there is much diversity among people living with dementia, including younger people diagnosed with the condition. The relevance of the aging body would not be so absolute in such instances. Furthermore, might a reliance upon therapy risk the ongoing medicalisation of dementia, a paradigm that has been challenged by established dementia scholarship included within this article [6,7,8,12]? Better, perhaps, to follow the lead of Burke and Jones [39], who advised that lay people, well briefed, could offer effective opportunities for engagement in physical activity. This could maximise the chance individuals have to participate on an everyday basis, as advocated here. What place within this for family carers? This article has been largely silent on their situation and potential to contribute, focusing the narrative upon the persons themselves. Looking ahead, this is a deficit that requires addressing because, as has been shown, relationships are crucial within the dementia context, and there are no more significant relationships than between those living with dementia and family carers. What role too for people living with dementia themselves in the facilitation of activity? The section exploring collaboration made clear the value of sharing agency and power. More clarity is required about how people themselves can expect to contribute.

Finally, where should physical activity be offered so that people living with dementia gain the most from it? Bearing in mind the preceding discussion, a default answer is likely to be: everywhere feasible within the context of everyday life. That is reasonable and sensible, but places will include hospitals and care/nursing homes. These are venues where people living with dementia spend time, but they are places where control over the physical environment is likely to be limited by constraints, including organisational ones. Leisure and fitness centres, similarly, would seem to offer the opportunity for everyday physical activity. They are under threat from challenges to their resources though [46,47], and work has only just started considering how those spaces can best be used to afford opportunities for good-quality physical activity for people living with dementia [44]. Thus, to answer the question what should happen next, there remains much to consider.

## 5. Conclusions

This article has highlighted ways in which physical activity can offer more to people living with dementia beyond the physical (important though that is). It has set out ways in which physical activity potentially presents individuals with the wherewithal to sustain their place in the world. To do so, it drew upon scholarship from the dementia field that promotes person-centred approaches to care and support and encouraged consideration of this within the milieu of physical activity. It went further, however. By emphasising the significance of human rights and social citizenship approaches to dementia, the article made the case that physical activity should not be seen as something that is nice and of potential value but as an essential element of everyday life for people living with dementia.

What should happen next is uncertain, as evidenced by the questions posed in the preceding section. What is clear, however, is that physical activity has much to offer to people living with dementia, and at present, its full potential is not being realised. Whether this is because of lack of knowledge, confidence or resources may be uncertain, but the priority must be to consider what is articulated here and use it to enhance the opportunities people living with dementia have to engage in physical activity.

## Data Availability

Data supporting the reported results can be found in the references, which are set out below.
